# A novel underwater endoscopic submucosal dissection via continuous irrigation method for esophageal squamous cell carcinoma with severe fibrosis

**DOI:** 10.1055/a-2601-0243

**Published:** 2025-06-13

**Authors:** Takahiro Muramatsu, Masakatsu Fukuzawa, Sakiko Naito, Midori Mizumachi, Satoshi Shimai, Takashi Yorozu, Takao Itoi

**Affiliations:** 1 38548 Department of Gastroenterology and Hepatology, Tokyo Medical University Hospital, Tokyo, Japan; 2 38548 Department of Diagnostic Pathology, Tokyo Medical University Hospital, Tokyo, Japan

The usefulness of underwater endoscopic submucosal dissection (UESD) has been previously reported 1 2 . Underwater procedures provide a clear endoscopic view without halation and facilitate lesion dissection by buoyancy. However, underwater conditions often result in poor endoscopic views due to intestinal residue, bleeding, and air bubbles caused by resection. Gel immersion 3 , gas-free immersion 4 , and bubble-free UESD 5 have been reported as countermeasures; however, methods to improve the visual field have not been established. Herein, we report a case of esophageal cancer with fibrosis that was resected by UESD using a novel continuous irrigation method (CIM) that blows bleeding and bubbles while providing a water pressure effect ( Video 1 ). The patient was a 68-year-old man who had undergone ESD for esophageal cancer two years prior. Surveillance endoscopy revealed esophageal cancer (18 mm, type 0–IIa) on the post-ESD scar ( Fig. 1 ). UESD was performed due to extensive scarring and severe fibrosis on the proximal side of the lesion. When a mucosal incision was made on the distal side of the lesion, the visual field was poor because of bleeding and bubbles ( Fig. 2 **a–c** ). Therefore, CIM ( Fig. 3 **a, b** ) was used to blow the bleeding and bubbles to obtain a clear field of view ( Fig. 2 **d–g** and Fig. 3 **c–f** ). Submucosal dissection was performed via CIM after a circumferential incision was made. The CIM also provided a water pressure effect and facilitated the approach to the fibrotic submucosal layer, resulting in successful *en bloc* resection ( Fig. 2 **h–l** ). The pathological diagnosis was curative resection ( Fig. 4 ). CIM helps overcome the disadvantages of UESD, such as a poor visual field due to bleeding and bubbles. It also helps overcome severe fibrosis due to the sustained water pressure effect. This simple method ensures a constant endoscopic field of view and allows the smooth continuation of UESD.

**Fig. 1:**
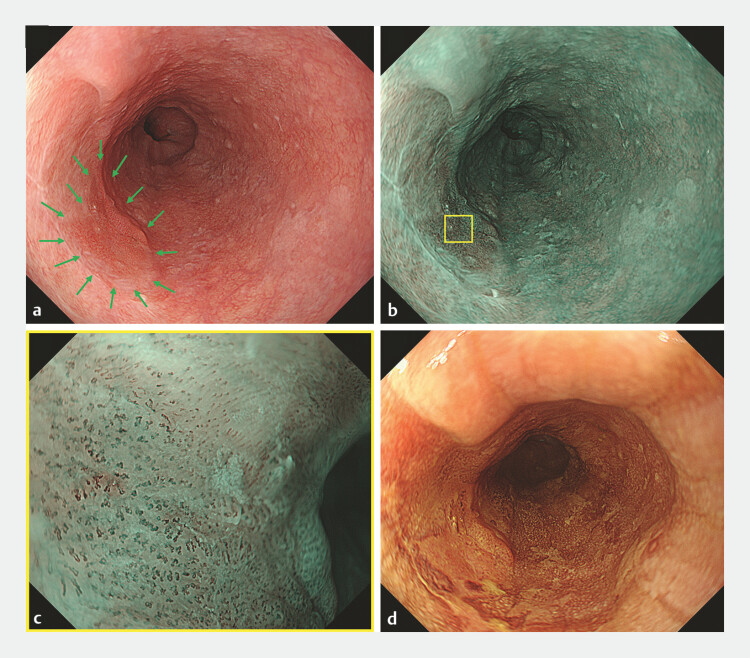
Endoscopic image. **a** White light image. Upper gastrointestinal endoscopy revealed a flat, elevated lesion (18 mm, type 0–IIa) located on the scar after endoscopic submucosal dissection in the middle thoracic esophagus. **b** NBI view. The lesion was visible as a brownish area. **c** Magnified NBI view of the yellow square in **b** . The loop vessels were observed to have dilatation, tortuosity, caliber changes, and various shapes corresponding to the Japan Esophageal Society Classification type B1. **d** The lesion was visible as the unstained area in endoscopic iodine staining. Abbreviation: NBI, narrowband imaging.

**Fig. 2:**
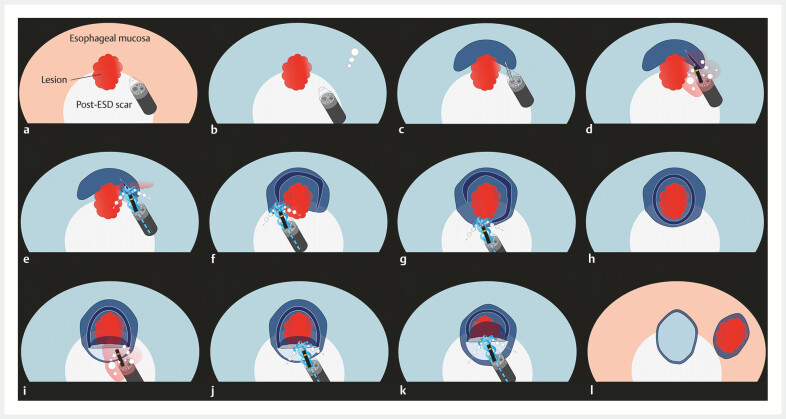
Schema of UESD with CIM for esophageal cancer with severe fibrosis. **a** Under gas view. **b** Underwater view. **c** Local injection was performed. **d** A mucosal incision was made on the distal side of the lesion. Bleeding and bubbles associated with the incision resulted in a poor endoscopic view. **e** Bleeding and bubbles were blown out using the CIM and the field of view became clear. **f** The mucosal incision was widened using CIM. **g** The mucosal incision of the proximal side also performed via CIM. **h** A complete circumferential incision was created. **i** During performing submucosal dissection, the bleeding and bubbles also occurred. **j** CIM clarified the visual field and submucosal dissection was performed. **k** The CIM also creates water pressure effects that support submucosal dissection for severe fibrosis. **l** Complete en bloc resection is achieved. Abbreviations: CIM, continuous irrigation method; UESD, underwater endoscopic submucosal dissection.

**Fig. 3:**
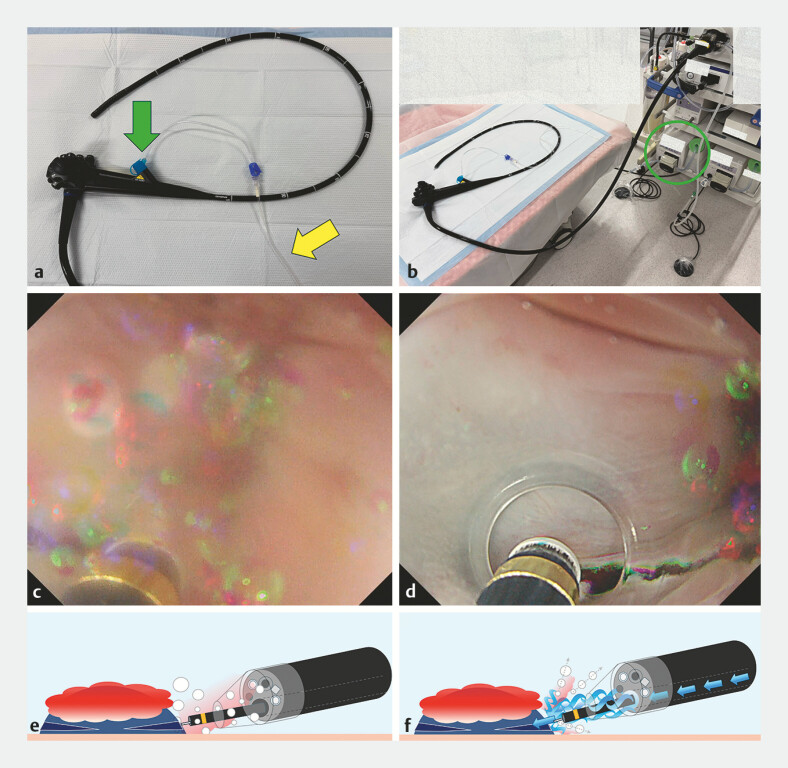
Setting of CIM with endoscopic view and schema. **a** A dedicated BioShield irrigator (green arrow) and 100 cm extension tube (yellow arrow) are required for the CIM. **b** Connecting the extension tube to the endoscopic flushing pump (green circle). **c** Endoscopic view of a normal UESD. Bleeding and air bubbles entering the tip hood result in poor vision. **d** Endoscopic view of the UESD with CIM. Bleeding and air bubbles were blown out of the hood, and the field of view became clearer. **e** Schema of normal UESD. **f** Schema of UESD with CIM. Abbreviations: CIM, continuous irrigation method; UESD, underwater submucosal dissection.

**Fig. 4:**
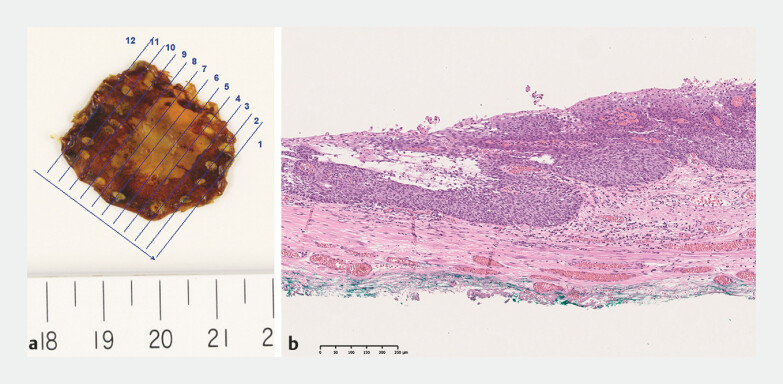
Macroscopic and histopathological images of the resected specimen. **a** Macroscopic image of the specimen. **b** Histopathological image of the specimen. The pathological diagnosis was squamous cell carcinoma in the lamina propria mucosae without lymphovascular invasion and negative margins.

Novel underwater endoscopic submucosal dissection via continuous irrigation for esophageal squamous cell carcinoma with severe fibrosis.Video 1

Endoscopy_UCTN_Code_TTT_1AO_2AG_3AD

## Notes

Endoscopy E-Videos https://eref.thieme.de/e-videos
*E-Videos* is an open access online section of the journal *Endoscopy* , reporting on interesting cases and new techniques in gastroenterological endoscopy. All papers include a high-quality video and are published with a Creative Commons CC-BY license. Endoscopy E-Videos qualify for HINARI discounts and waivers and eligibility is automatically checked during the submission process. We grant 100% waivers to articles whose corresponding authors are based in Group A countries and 50% waivers to those who are based in Group B countries as classified by Research4Life (see: https://www.research4life.org/access/eligibility/ ). This section has its own submission website at https://mc.manuscriptcentral.com/e-videos .
